# Navigating Complexities in Pediatric Rhabdomyosarcoma: A Case Report on Its Neurological Implications

**DOI:** 10.7759/cureus.45991

**Published:** 2023-09-26

**Authors:** Anum Haider, Vyshnavidevi Sunkara, Sagarika Basnet, Maryam Affaf, Chukwuyem Ekhator

**Affiliations:** 1 Internal Medicine, Bahria University Medical and Dental College, Karachi, PAK; 2 Medicine, Katuri Medical College, Guntur, IND; 3 Internal Medicine, Kathmandu Medical College, Kathmandu, NPL; 4 Internal Medicine, Women’s Medical and Dental College, Abbotabad, PAK; 5 Neuro-Oncology, New York Institute of Technology, College of Osteopathic Medicine, Old Westbury, USA

**Keywords:** medicine, understanding and treating pain paediatrics, oncology, primary rhabdomyosarcoma, rhabdomyosarcoma

## Abstract

This case report presents a 12-year-old male with a rare manifestation of rhabdomyosarcoma (RMS), emphasizing diagnostic and therapeutic challenges. The patient exhibited firm, tender facial swelling and underwent diagnostic procedures including imaging and biopsy, confirming RMS. Treatment involved a multi-agent chemotherapy regimen and radiotherapy, leading to a significant tumor reduction. However, neurological deficits emerged one month after treatment, suggesting neural invasion. The case highlights the need for vigilant monitoring and a multimodal treatment approach in managing RMS. It also raises questions about neural invasion risks post-treatment, contributing valuable insights to existing literature and advocating for further research in this rare pediatric cancer.

## Introduction

Rhabdomyosarcoma (RMS) is a relatively rare form of cancer that predominantly affects soft tissues, particularly skeletal muscle tissue, although it can also manifest in hollow organs such as the bladder or uterus [1]. This malignancy is most commonly diagnosed in children and adolescents, making it a significant concern in pediatric oncology [2].

The origin of RMS can be traced back to rhabdomyoblasts, which are precursor cells to skeletal muscle. Genetic alterations in these cells can inhibit their normal development, leading to the formation of tumors [3]. While the exact etiology remains elusive, factors such as genetic predisposition and family history are thought to contribute to the risk of developing RMS.

Clinically, RMS often presents in the head and neck region or the urinary system. Symptoms can vary widely depending on the tumor's location and may include headaches, swollen eyes, and bleeding [4]. Treatment modalities for RMS typically encompass a combination of radiation, chemotherapy, and surgical intervention. One of the critical challenges in managing RMS is the potential for metastasis, particularly to the lungs and bones, necessitating vigilant monitoring and ongoing care [5].

## Case presentation

**Figure 1:**
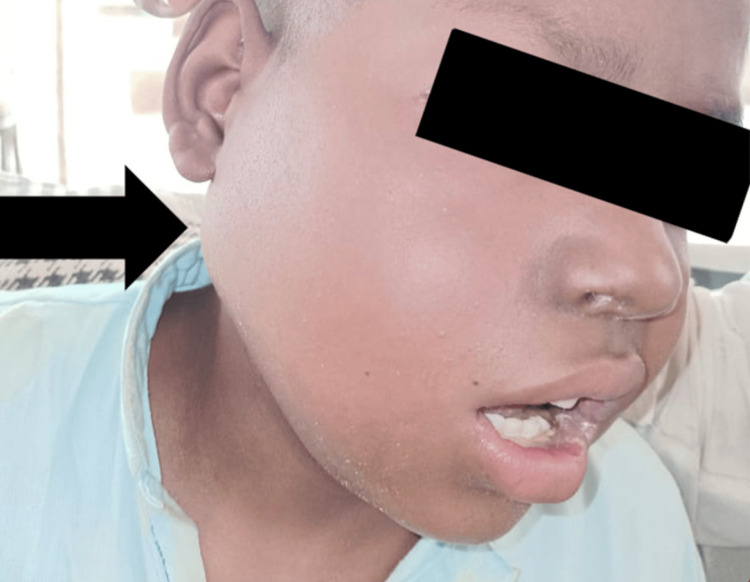
Unilateral facial mass located below the angle of the mandible on the right side, measuring 8 cm x 5 cm

A 12-year-old male presented to the hospital with a unilateral facial mass located below the angle of the mandible on the right side. The mass had been present for approximately 18 months. It was non-fluctuant, non-mobile, and tender to touch, measuring 8 cm x 5 cm (Figure 1). The patient experienced trismus and reported pain radiating to the right periorbital region. Additionally, he complained of persistent frontal headaches that were unresponsive to pharmacological intervention. Leukocoria of the right eye was also noted, having developed over the past three months.

Initially, the patient sought medical advice from a local physician, who referred him to a dental specialist. The dental examination raised suspicions of a malignant neoplasm, prompting a referral to an oncology center for further evaluation. Initially, a complete blood workup was carried out, including a complete blood count, liver function tests, and renal function tests. The results were unremarkable, except for the hemoglobin level, which was 10.1 g/dl. Diagnostic imaging via CT scan of the head and neck revealed a heterogeneous, lobulated soft tissue density mass in the right parotid and masticator spaces. The mass extended into the parapharyngeal and carotid spaces without vascular encasement. Superior extension into the infratemporal fossa was noted, with medial bulging causing luminal narrowing in the nasopharynx and oropharynx. Erosion of both pterygoid plates and scalloping of the adjacent mandible were also observed.

**Figure 2:**
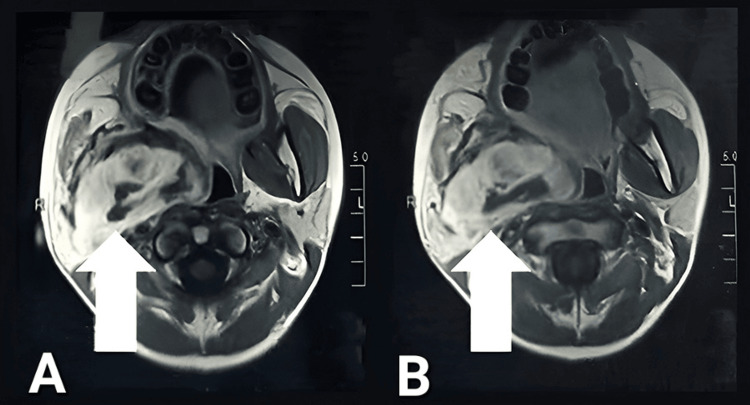
The MRI of the head showing a heterogeneous, lobulated soft tissue mass in the right parotid and masticator spaces, as marked by arrows in panels A and B.

**Figure 3:**
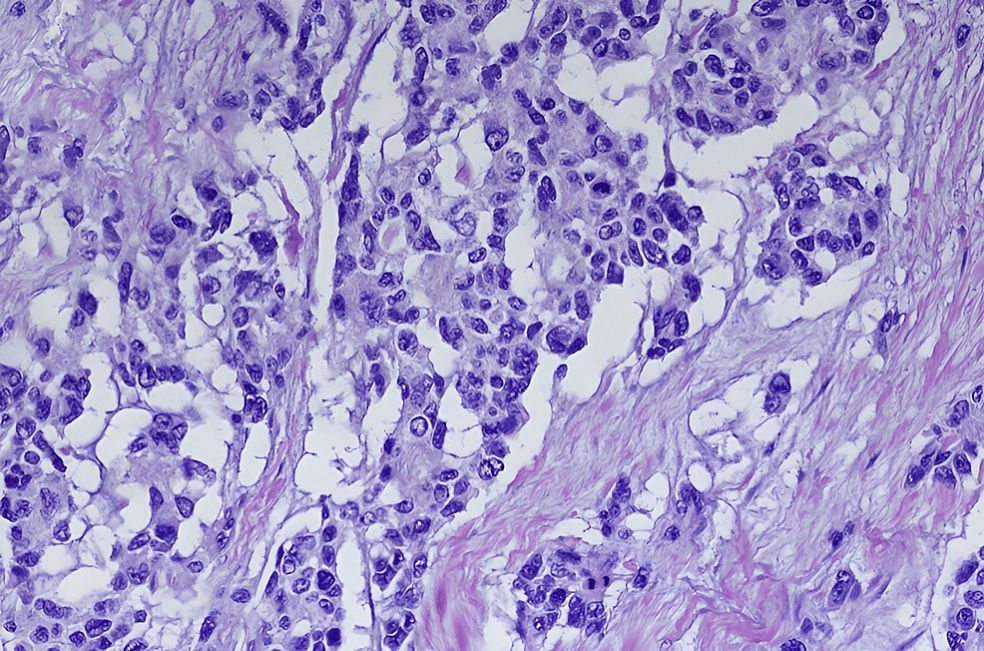
H&E staining of the biopsy sample revealing cells that are round to oval with hyperchromatic nuclei and cytoplasmic clearing H&E: Hematoxylin and eosin

The MRI findings corroborated these observations, revealing heterogeneous abnormal MR signal intensities in multiple regions, including the right masticator and parapharyngeal and paranasopharyngeal spaces. Enlarged cervical lymph nodes were identified bilaterally at levels 1a and 1b, with the largest node on the right side measuring 9.3 mm x 7.3 mm (Figure 2). No vascular abnormalities were detected. Hematoxylin and eosin (H&E) staining of a biopsy sample revealed a neoplasm infiltrating the skeletal muscle (Figure 3). The cells were round to oval with hyperchromatic nuclei and cytoplasmic clearing. Immunohistochemical staining for desmin was positive, supporting a diagnosis of RMS.

**Figure 4:**
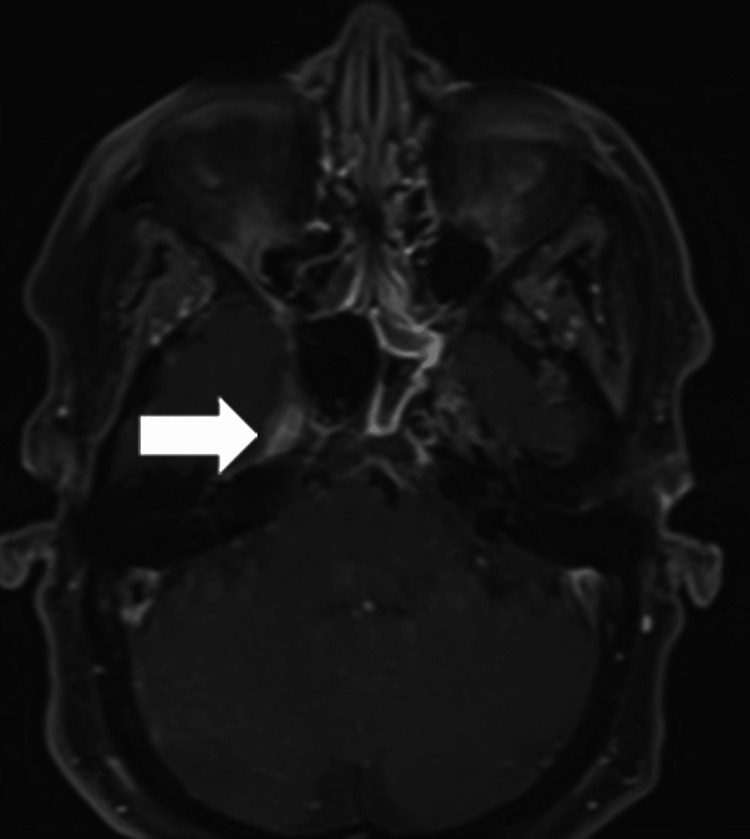
RMS with neural invasion The tumor can be seen extending into cavum trigeminale and adjacent areas. RMS: Rhabdomyosarcoma

The patient was diagnosed with RMS of the embryonal subtype following a series of diagnostic tests. The initial chemotherapy regimen consisted of intravenous (IV) vincristine, actinomycin, and cyclophosphamide, as decided by a multidisciplinary team. This was followed by a course of radiation therapy, both of which contributed to a reduction in tumor size. However, one month after treatment, the patient began experiencing severe headaches and neurological deficits, including diminished reflexes and motor function in the left upper and lower extremities. A repeat MRI confirmed these symptoms to be a result of neural invasion by the tumor (Figure 4).

The patient is currently on a comprehensive IV treatment regimen that includes dactinomycin, cyclophosphamide, vincristine, pheniramine maleate, ondansetron, corticosteroids, and other chemotherapeutic agents for symptomatic and conservative management. Concurrently, a complete blood count, liver function tests, and renal function tests have shown no abnormalities. Future therapeutic strategies will be determined upon the completion of the ongoing chemotherapy regimen.

## Discussion

Rhabdomyosarcoma accounts for approximately 3% of all pediatric malignancies, categorizing it as a relatively rare form of cancer. The highest incidence rates are observed among children aged one to four years, followed by those in the 10 to 14 and 15 to 19 age groups that exhibit lower frequencies [6]. The age of our patient (12 years) thus presents an atypical case within the RMS demographic.

Oral RMS manifests not only localized oral symptoms but also systemic signs. Common symptoms include trismus, making it difficult to fully open the mouth or comfortably chew and swallow food. Additionally, patients may experience numbness or paresthesia due to nerve involvement, as well as altered speech and breathing difficulties when tumors are located in the soft palate, tongue, or throat [4]. In alignment with these clinical features, our patient also exhibited trismus, speech difficulties, and neurological deficits, including loss of power and reflexes in the left arm and leg.

Rhabdomyosarcoma, a cancerous tumor of muscle origin, has several subtypes with unique features. Embryonal rhabdomyosarcoma (ERMS) is most common in young children and often appears in the head or genitourinary area, with a generally better prognosis. Alveolar rhabdomyosarcoma (ARMS) usually affects older kids and is often found in limbs, with a less favorable outlook. Other rarer forms include pleomorphic and spindle-cell variants. Treatment strategies vary by subtype and may include surgery, chemotherapy, and radiation.

Chemotherapeutic intervention is a cornerstone in the management of RMS. A multi-agent regimen, often tailored to the patient's risk profile, is commonly employed. Drugs such as dactinomycin, vincristine, and cyclophosphamide are administered over a course ranging from six months to a year, either orally or intravenously [7]. In the context of our patient, a comprehensive treatment plan was implemented that included both chemotherapy and radiotherapy, leading to a significant reduction in tumor size.

Despite the initial success in reducing the tumor size, the patient began experiencing severe neurological deficits one month after completing treatment. A repeat MRI was conducted to investigate these symptoms, confirming neural invasion by the tumor. These developments not only underscore the aggressive nature of RMS but also highlight the need for vigilant monitoring and possibly supplementary adjuvant therapies. The patient's experience is consistent with established studies on the effectiveness of these drug combinations in RMS management. However, it also brings attention to the complexity and challenges involved in treating this condition. Compared to similar cases reported in the literature, our case suggests that while chemotherapeutic regimens are generally effective in reducing tumor size, they may not be sufficient to prevent complications such as neural invasion or metastasis.

The complexities and challenges presented by our case offer valuable insights for future research and clinical practice in the management of RMS. Specifically, the occurrence of neurological deficits post-treatment raises questions about the potential for neural invasion, even after significant tumor reduction. This could prompt further studies on the mechanisms of neural invasion in RMS and the development of targeted therapies to prevent such complications. Additionally, our case highlights the need for long-term, vigilant monitoring of patients undergoing chemotherapeutic regimens, suggesting that future clinical guidelines could benefit from incorporating more robust follow-up protocols. Given the aggressive nature of RMS, research into novel adjuvant therapies and more personalized treatment plans based on genetic or molecular markers could also be an avenue for future studies.

## Conclusions

This case report details a rare presentation of RMS in a 12-year-old, diverging from the typical age group most affected by this malignancy. Despite initial success with chemotherapy and radiotherapy, the patient experienced neurological complications, emphasizing the aggressive and invasive nature of the disease. The case underscores the critical need for early diagnosis, vigilant monitoring, and a multimodal treatment approach. Given the complexities and challenges presented, this case adds valuable insights to the existing literature and calls for ongoing research to optimize treatment strategies for this rare pediatric cancer.
